# Irradiance driven trophic plasticity in the coral *Madracis pharensis* from the Eastern Mediterranean

**DOI:** 10.1038/s41598-024-54217-3

**Published:** 2024-02-13

**Authors:** Gretchen Goodbody-Gringley, Stephane Martinez, Jessica Bellworthy, Alex Chequer, Hagai Nativ, Tali Mass

**Affiliations:** 1 Reef Ecology and Evolution, Central Caribbean Marine Institute, Little Cayman, Cayman Islands; 2 Department of Marine Biology, Leon H. Charney School of Marine Sciences, University of Haifa, Haifa, Israel; 3 Leon H. Charney School of Marine Sciences, Morris Kahn Marine Research Station, University of Haifa, Haifa, Israel

**Keywords:** Mesophotic, Photophysiology, Stable Isotopes, Morphology, Coral symbiosis, Marine biology, Ecophysiology, Ecophysiology

## Abstract

The distribution of symbiotic scleractinian corals is driven, in part, by light availability, as host energy demands are partially met through translocation of photosynthate. Physiological plasticity in response to environmental conditions, such as light, enables the expansion of resilient phenotypes in the face of changing environmental conditions. Here we compared the physiology, morphology, and taxonomy of the host and endosymbionts of individual *Madracis pharensis* corals exposed to dramatically different light conditions based on colony orientation on the surface of a shipwreck at 30 m depth in the Bay of Haifa, Israel. We found significant differences in symbiont species consortia, photophysiology, and stable isotopes, suggesting that these corals can adjust multiple aspects of host and symbiont physiology in response to light availability. These results highlight the potential of corals to switch to a predominantly heterotrophic diet when light availability and/or symbiont densities are too low to sustain sufficient photosynthesis, which may provide resilience for corals in the face of climate change.

## Introduction

In contemporary tropical and subtropical oceans, symbiotic corals provide a literal and figurative ecological framework that retains nutrients, supports high rates of primary production, and permits extensive biological diversity. However, these fragile ecosystems are threatened with extinction in the coming century^1–3^. One of the dominant threats to reefs is the loss of autotrophic symbionts or dysbiosis as a result of increasing seawater temperatures^4,5^. Prolonged periods of dysbiosis may ultimately lead to coral mortality as many species are reliant on translocated photosynthate from endosymbiotic dinoflagellates in the family *Symbiodiniacaea* to meet the majority of their metabolic demands^5–8^. Yet, corals are known to exhibit adaptations to environmental gradients, such as alterations in *Symbiodiniacaea* species association and/or density^9–11^, feeding strategy^12^, and metabolism^13^. Conspecific corals, for example, typically exhibit an increase in symbiont density and chlorophyll concentration with increasing depth to maintain stable rates of photosynthesis under decreasing light availability^14–19^. On the other hand, some symbiotic corals living in extremely low light environments, such as caves and overhangs, maintain very few photosymbionts, appearing white in color^20^. In fact, not all calcifying corals have an obligate symbiosis, with several species expressing a facultative relationship where autotrophic symbionts may be absent or maintained at low densities in a non-stressed state^21^. These species often have sub-tropical distributions and are frequently found with reduced symbiont densities in low light conditions related to latitude, seasonality, or physical environmental parameters^22–24^. Facultative symbiosis might be advantageous for coral survival under future climate change scenarios, as higher densities of symbionts were found to result in increased bleaching severity^25^. Thus, understanding how facultative species function with variable symbiont densities will provide insight into the mechanisms that corals may employ during extended periods of dysbiosis^24^.

The genus *Madracis* is ubiquitous across coral reef habitats from shallow to deep regions, and includes obligate and facultative symbiotic species, as well as non-symbiotic species. The species *M. pharensis* has a broad distribution ranging from the Caribbean to the Atlantic, and into the Mediterranean Sea, at depths from 0 to 80 m^20^. In the Mediterranean, *M. pharensis* forms small, knobby colonies that have a facultative symbiosis with *Breviolum psygmophilum*^26^. The species is characterized by high morphological plasticity and wide environmental tolerance^27^, forming massive and encrusting colonies in cryptic sites compared to nodular colonies in the light and is commonly found without symbionts in caves^20^. However, while *M. pharensis* is documented to associate with *B. psygmophilum* in the Mediterranean, Frade et al.^11^ found differences in symbiont species associations across depth among congenerics. The species is therefore an excellent candidate to examine the connection between phenotype and physiology, and to explore mechanisms of adaptation by a facultative symbiotic coral to variable environmental conditions.

Examining coral photophysiology in situ primarily relies on instantaneous measurements such as maximum quantum yield which is commonly used as a proxy for photosynthetic efficiency^28^. However, the coral host itself can also attain nutrients via heterotrophy and thus the photosynthate produced by endosymbiotic *Symbiodiniacaea* is not the only available source of nutrients^29^. Importantly, previous studies have shown plasticity in reliance on autotrophy versus heterotrophy by facultative symbiotic corals, where conspecific individuals that typically rely on autotrophy switch to heterotrophy in less-favorable conditions^12,29,30^. One technique used to examine changes in photophysiology is chlorophyll variable fluorescence, which provides a wide range of measurements for fluorescent and photosynthetic parameters of an organism^31^. The diving-Fluorescence Induction and Relaxation (Diving-FIRe) fluorometer developed by Gorbunov & Falkowski^32^ uses a two-phase approach of both strong short pulses (induction phase) and weak modulated light (relaxation phase) to measure the steady state quantum yield of photochemistry in PSII (F_v_’/F_m_’), the functional absorption cross-section of PSII (σ_PSII_’; A^2^), the connectivity parameter (p) that determines the probability of excitation energy transfer between individual photosynthetic units, and the maximum photosynthetic rate (P_max_: electron·s^−1^·PSII^−1^).

In contrast to other fluorescence techniques that are amplitude-based^33–35^, the FIRe combines both classic amplitude-based analysis and a new kinetic-based approach to directly measure the absolute value of light-driven electron flux in PSII (electron transport rates (ETR)). The amplitude-based model does not measure ETR directly but is based on the change in amplitude of chlorophyll fluorescence (ΔF_v_’/F_m_’) as a proxy of quantum yield of photochemistry in PSII under ambient irradiance^33^. On the other hand, kinetic analysis is based on monitoring the kinetics of the quinone reoxidation in PSII to quantify the photosynthetic ETR^36^. The ETR can then be converted to the rates of carbon fixation using the electron yield of carbon fixation^37^. Gorbunov and Falkowski^38^ revealed that the kinetic analysis offers more accurate ETR measurements, as evidenced by stronger correlation with growth rates (and thus net production), at least in high-light environments (e.g., shallow coral reefs).

In corals, such measurements of ETRs have additional advantages, as the kinetic analysis is not affected by the “pigment packaging” effect, which may be very strong in densely pigmented coral and can therefore be used to understand photochemistry under different environmental conditions in situ. To examine changes in food source and trophic level, the compound-specific Stable Isotope Analysis of Amino Acids (CSIA-AA) provides a powerful tool. Essential AA can be used to trace changes in the carbon sources and diet as they can only be synthesized by primary producers, and therefore do not change in carbon isotope value with trophic transfers^39^. Examining the source AA, phenylalanine, for which the nitrogen isotope does not change between trophic levels, and the trophic AA, glutamic-acid, which increases its nitrogen isotope value with every trophic transfer, can be used to calculate the trophic position (TP) as an indicator of an autotrophic (TP = 1), mixotrophic (TP = 1.5–2), or heterotrophic (TP > 2) diet^40^. Thus, combining photophysiology with stable isotope analyses provides a comprehensive picture of coral nutrient cycling.

**Figure 1:**
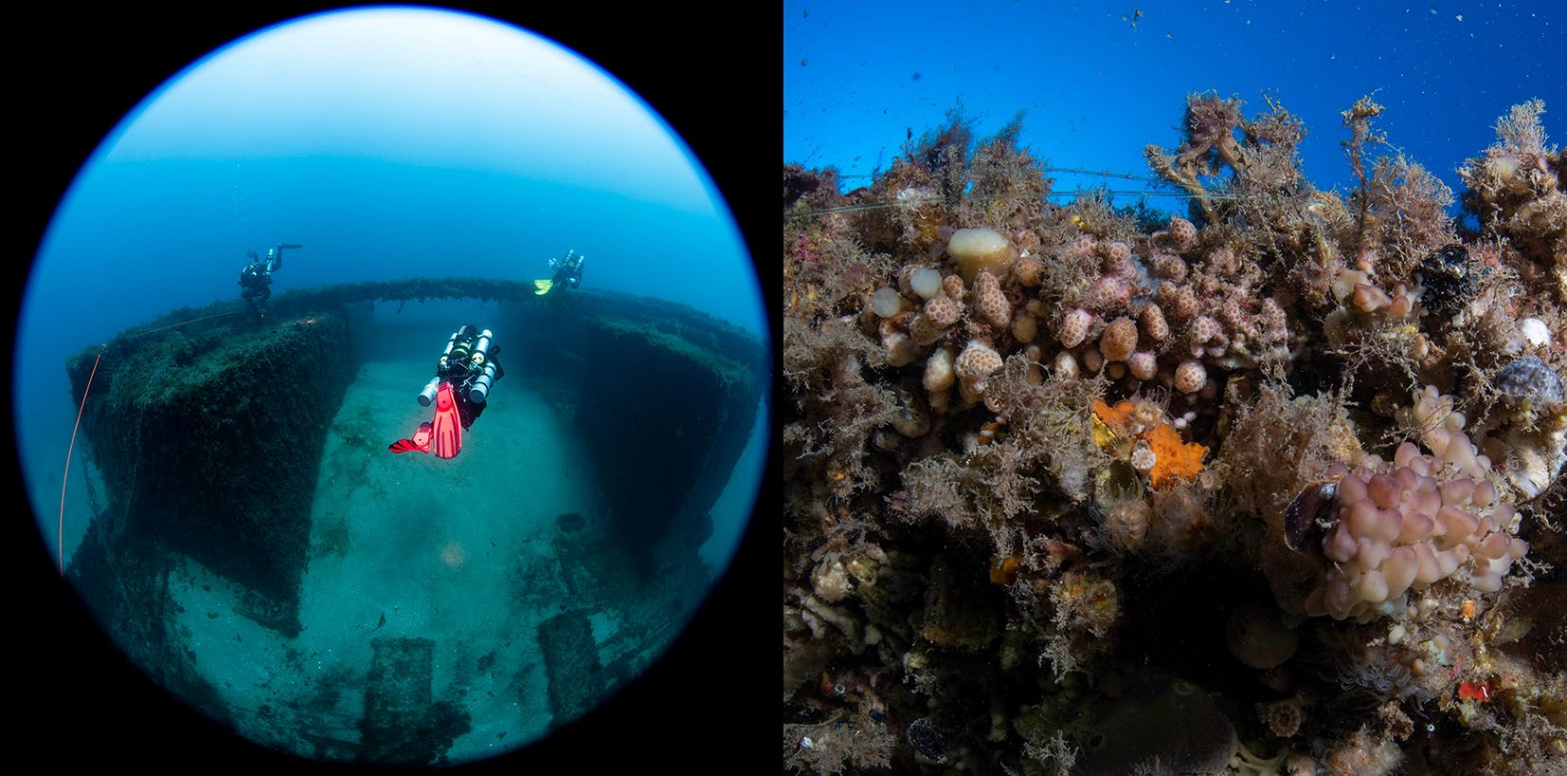
Image of divers on the top side of the Leonid shipwreck with an overview of the site (left). Image of the benthos on the side-facing surface of the wreck showing various species of algae, invertebrates, and stony corals (right).

**Figure 2:**
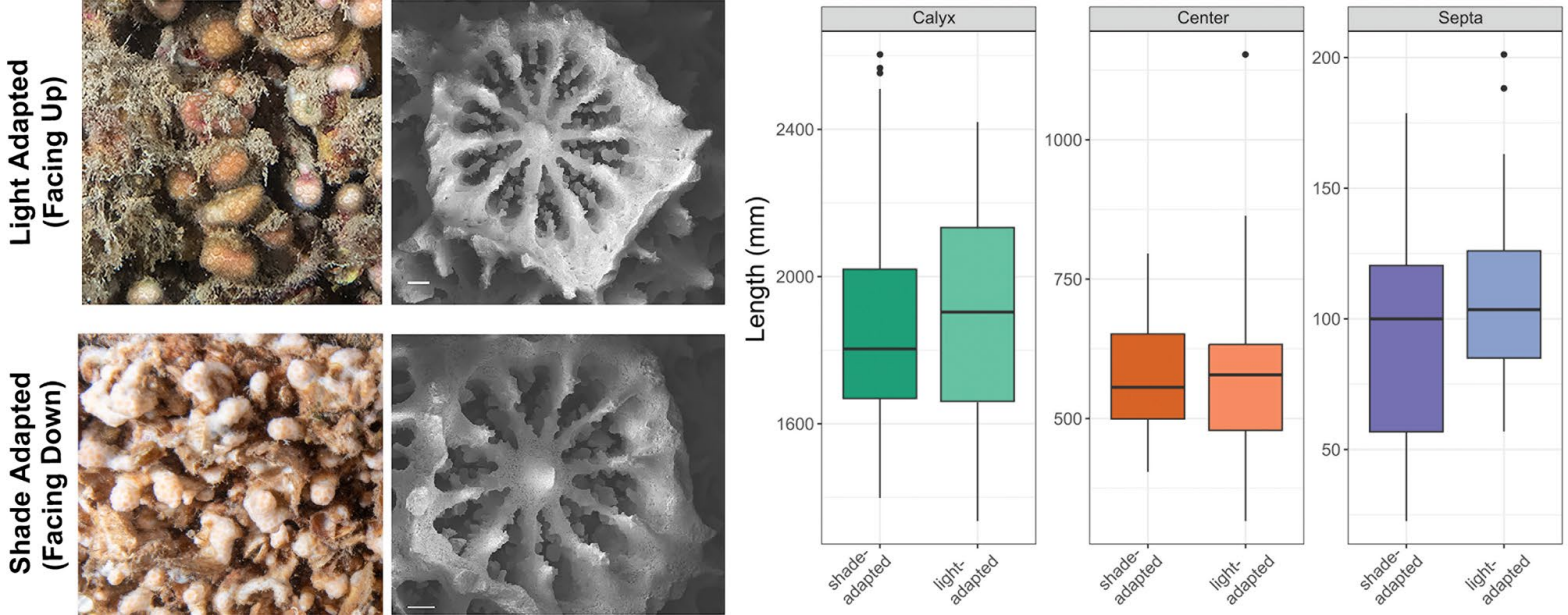
(Left) Representative corals found on the surface of the wreck oriented upwards towards the light (top left; light-adapted) and downwards towards the dark (bottom left; shade-adapted), with corresponding SEM images of individual polyps used for skeletal analyses. Scale bar 200 µm. (Right) Analyses of (**A**) calyx width, (**B**) center (columella) width, and (**C**) septa width based on SEM images. Horizontal black lines within boxes are median values and box limits represent first and third quartiles. Whiskers represent 1.5 times the interquartile range. Round black points are individual sample data.

In the Bay of Haifa, Israel, *M. pharensis* colonies inhabit the surface of the Leonid shipwreck at 30 m depth. These colonies can be found in two contrasting orientations that express different phenotypes, where colonies facing the light are generally brown in color, while those facing downwards in overhanging shaded compartments are pale pink or white (Figs. 1 and 2). The aim of this study was to compare these divergent coral phenotypes using a combination of photophysiology, nutrient acquisition, and skeletal morphology, to describe how these corals function under different light conditions. As light is a determinant in symbiotic-coral distribution and plays a critical role in coral bleaching, changes in photosynthetic efficiency, or potential photo acclimatization, are critical to understand how these organisms survive with reduced symbiont densities and thus how corals may function under future climate change scenarios.

## Results

**Figure 3:**
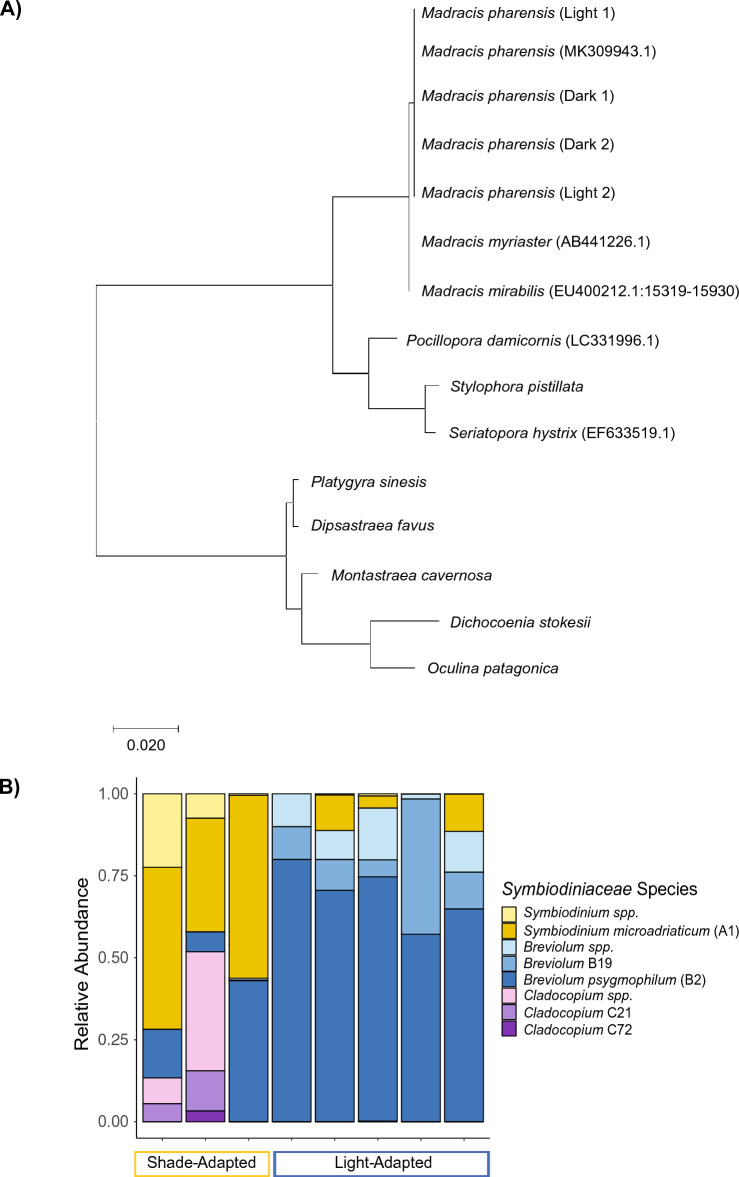
(**A**) Phylogenetic tree generated based on maximum likelihood and Tamura-Nei models of *COI* genetic sequences, including corals collected in the present study representing the different orientations (light and shade), known sequences of *M. pharensis*, and sequences from other closely related coral species. (**B**) Relative abundance of *Symbiodiniaceae* sequences, showing predominance of species in the genus *Breviolum* for light-adapted individuals compared to a mixed consortia of species including the genera *Symbiodinium, Breviolum,* and *Cladocopium* for shade-adapted individuals.

Measured skeletal features of individual polyps did not differ between light- and shade- adapted individuals (p > 0.05; Student’s T-test; Fig. 2). Analyses of cytochrome c oxidase subunit 1 (*CO1*) gene sequences using maximum-likelihood showed both light- and shade- adapted individuals fall within the same evolutionary branch as pooled *M. pharensis* samples confirming the species identity (Fig. 3A). Symbiont analysis of the internal transcribed spacer (*ITS2*) found the dominant symbiont in the light-adapted corals identified as *Breviolum psygmophilum* (clade B2), while the dominant symbiont in the shade-adapted individuals is *Symbiodinium microadriaticum*, clade A1 (Fig. 3B). Shade-adapted individuals were also found to host *Cladocopium* spp*.,* while light-adapted individuals were not.

**Figure 4:**
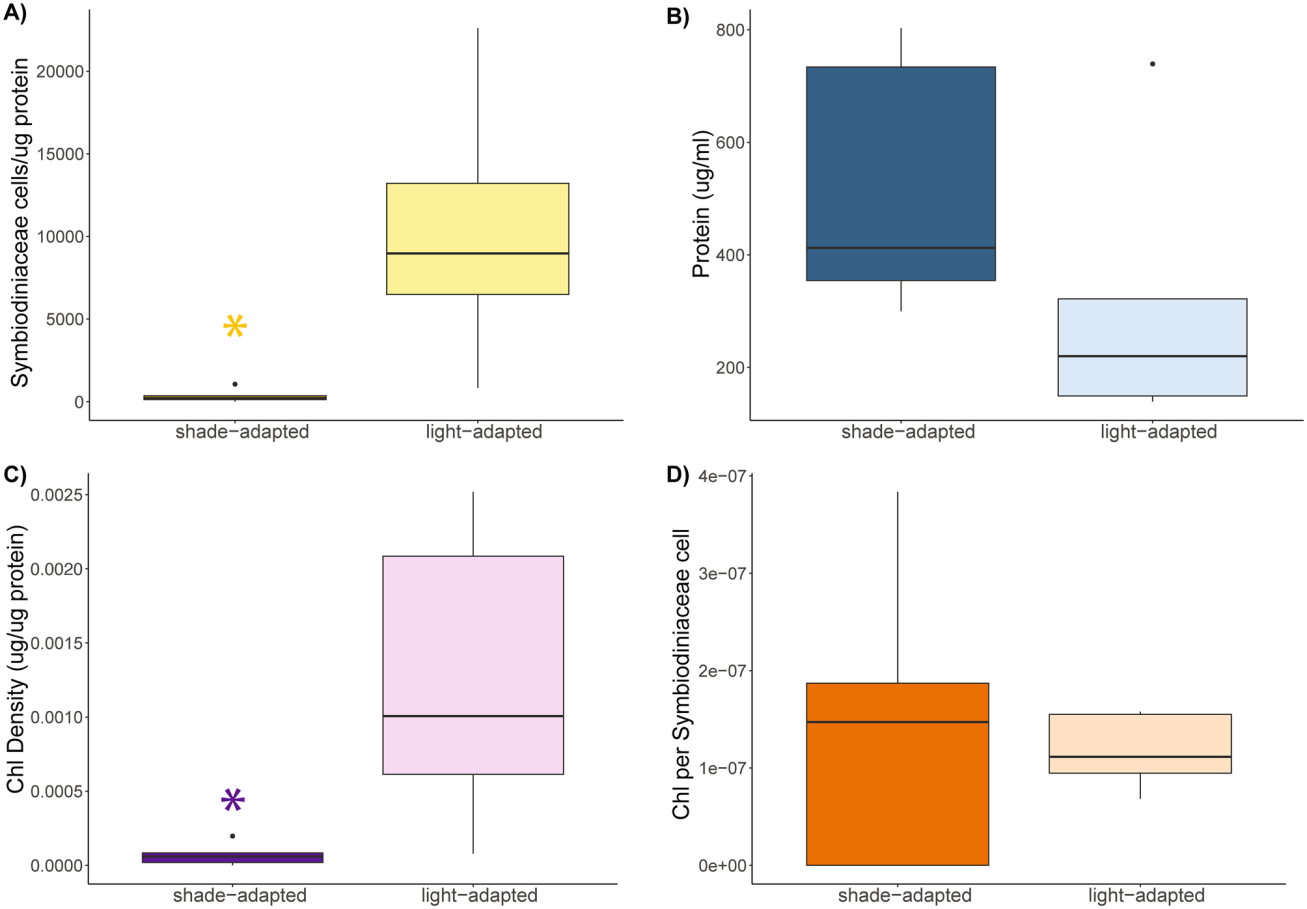
(**A**) Symbiont density per ug of protein, (**B**) density of protein per ml of coral tissue, (**C**) chlorophyll *a* density per ug of protein, and (**D**) chlorophyll density per symbiont cell based on orientation towards or away from the surface (light- vs. shade-adapted). Horizontal black lines within boxes are median values and box limits represent first and third quartiles. Whiskers represent 1.5 times the interquartile range.

Light-adapted individuals were found to have significantly higher density of photosymbionts and chlorophyll *a* concentration relative to total protein, compared to shade-adapted individuals (p = 0.013; p = 0.012; ANOVA; Fig. 4A and C), while chlorophyll density per symbiont cell was similar under both orientations (p > 0.05; ANOVA; Fig. 4D). Total protein content per ml of tissue was greater for shade-adapted individuals compared to light-adapted, however, this difference was not significant (p > 0.05; ANOVA; Fig. 4B).

**Figure 5:**
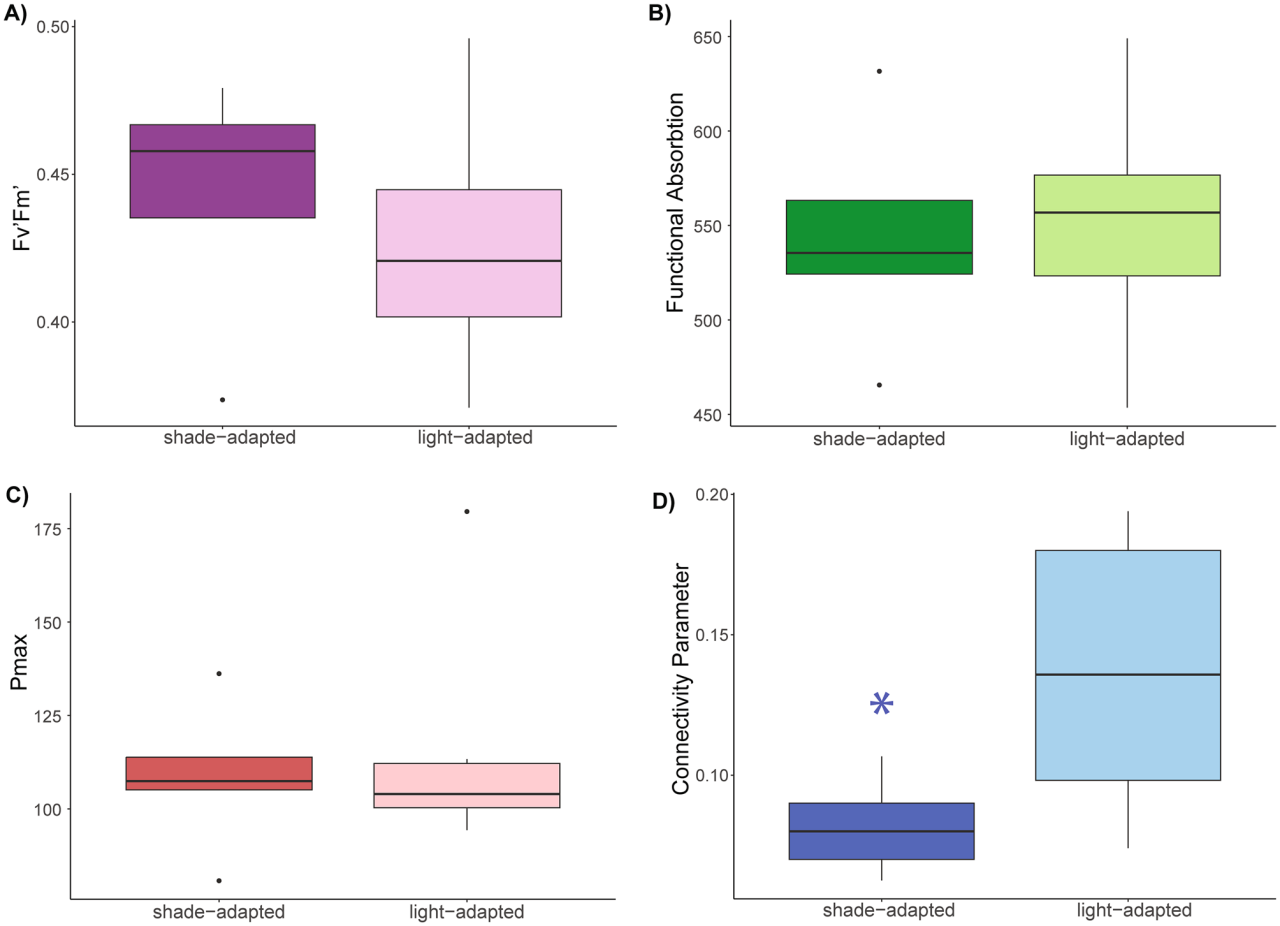
(**A**) Photosynthetic efficiency, (**B**) functional absorption, (**C**) maximum photosynthesis, and (**D**) connectivity parameter based on orientation towards or away from the surface (light- vs. shade-adapted). Horizontal black lines within boxes are median values and box limits represent first and third quartiles. Whiskers represent 1.5 times the interquartile range.

Photosynthetic efficiency, maximum photosynthetic yield, and the functional absorption of light did not differ among light-adapted and shade-adapted individuals (p > 0.05; ANOVA; Fig. 5A–C). However, the connectivity parameter was significantly lower in shade-adapted individuals compared to the light-adapted (p = 0.019; ANOVA; Fig. 5D).

**Figure 6:**
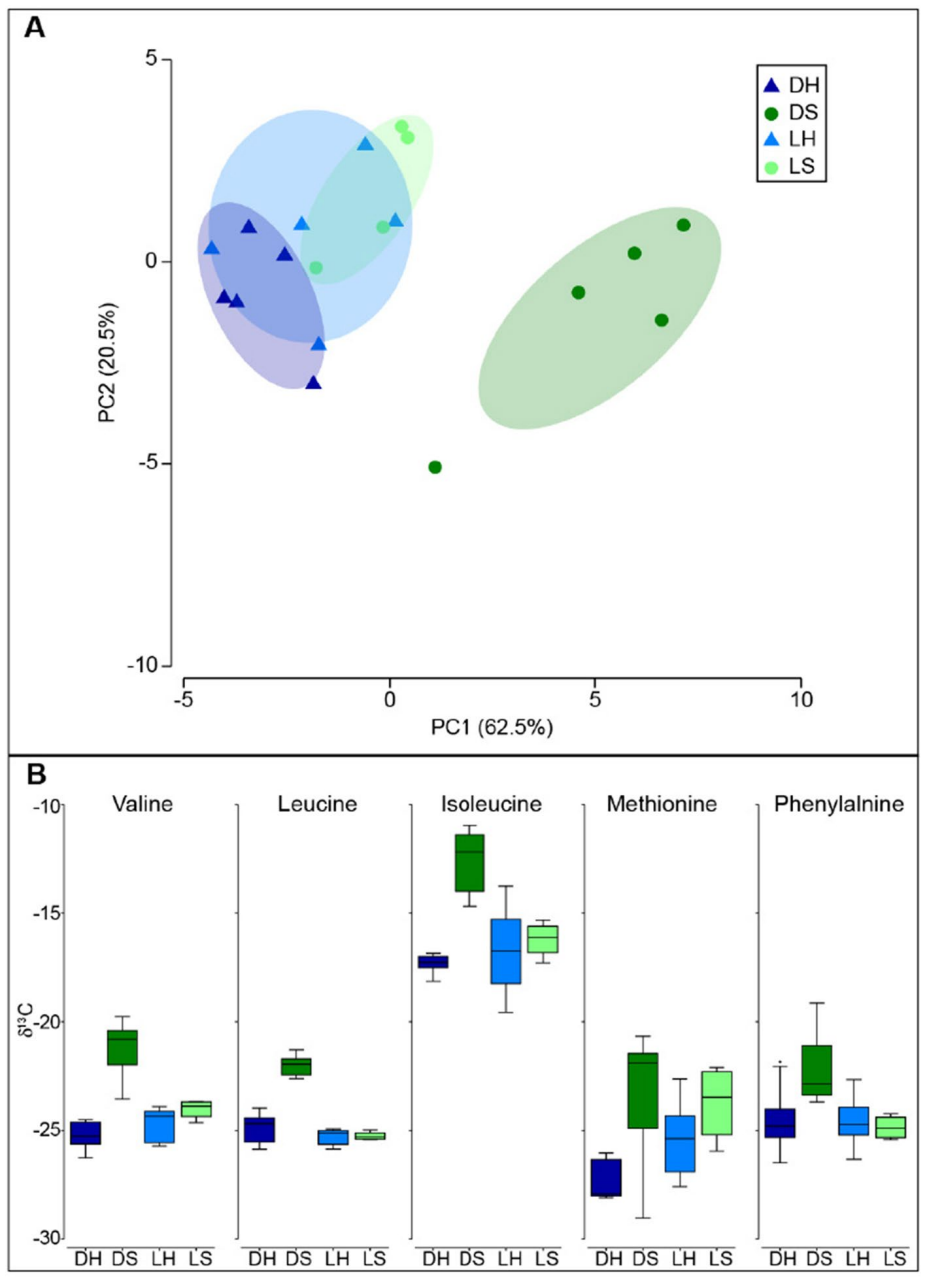
Carbon CSIA-AA of host and symbiont from corals that are light- and shade-adapted. (**A**) PCA of five essential amino acids (Valine, Leucine, Isoleucine, Methionine, and Phenylalanine). (**B**) Carbon isotopic values of five essential amino acids. Samples are shade-adapted host (DH, dark blue), shade-adapted symbiont (DS, dark green), light-adapted host (LH, light blue), and light-adapted symbiont (LH, light green).

**Figure 7:**
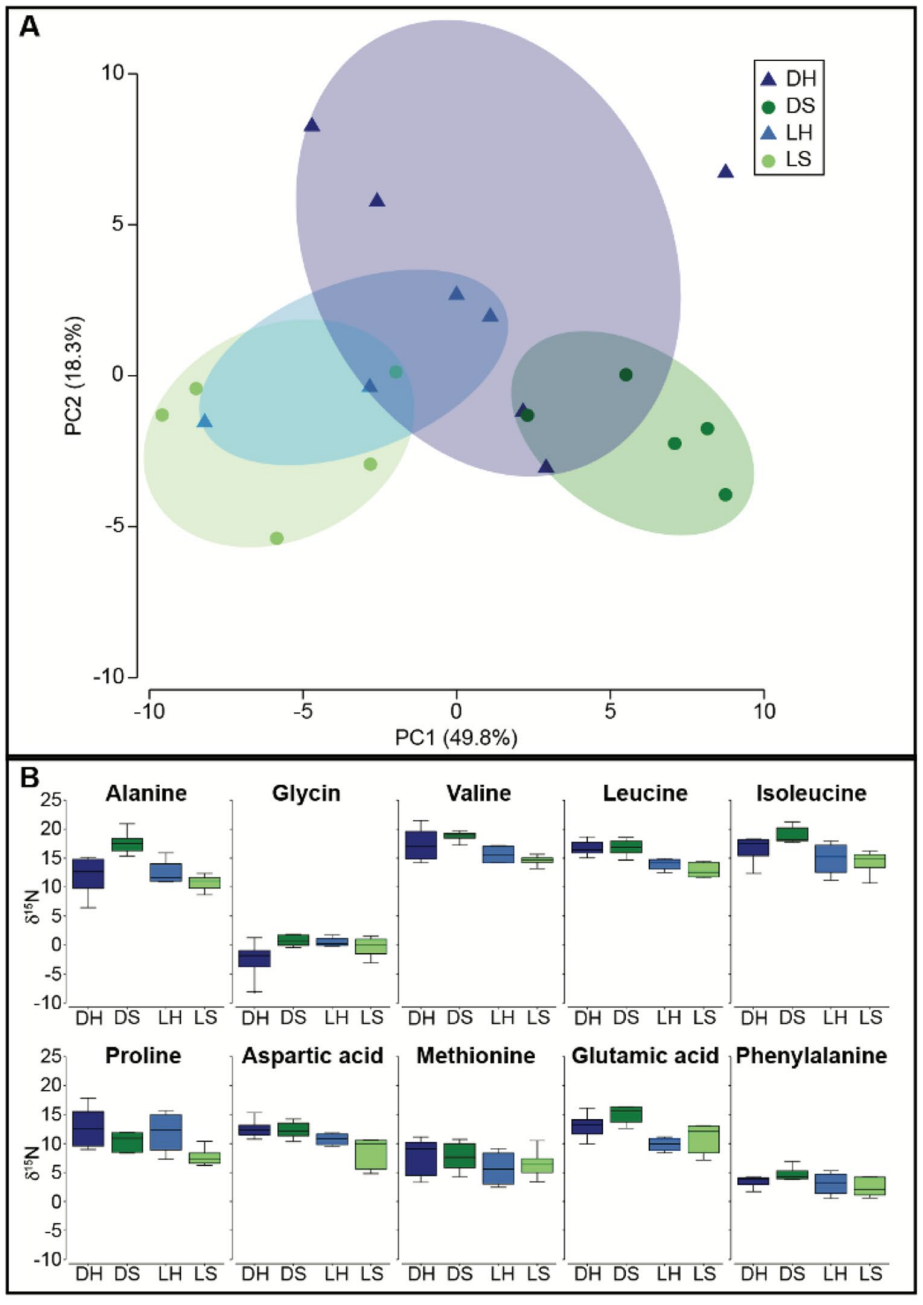
Nitrogen CSIA-AA of host and symbiont that are light- or shade-adapted. (**A**) PCA of ten amino acids (Alanine, Glycin, Valine, Leucine, Isoleucine, Proline, Aspartic acid, Methionine, and Phenylalanine). (**B**) Nitrogen isotopic values of ten amino acids. Samples are shade-adapted host (DH, dark blue), shade-adapted symbiont (DS, dark green), light-adapted host (LH, light blue), and light-adapted symbiont (LH, light green).

**Figure 8:**
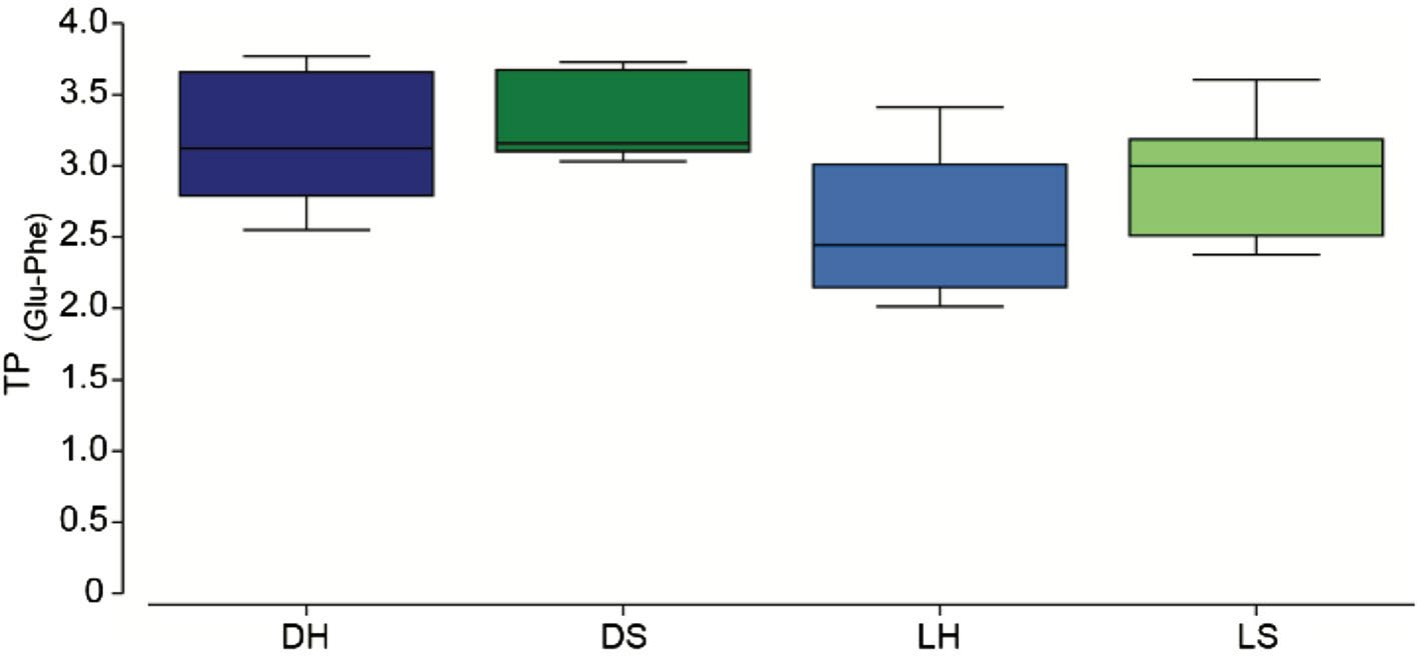
Glutamic acid and phenylalanine calculated trophic position of shade-adapted host (DH, dark blue), shade-adapted symbiont (DS, dark green), light-adapted host (LH, light blue), and light-adapted symbiont (LH, light green).

CSIA-AA analysis indicates that the carbon source for the shade-adapted symbionts was significantly different than that of the light-adapted host and symbionts (p = 0.008; PERMANOVA pairwise; Fig. 6). The carbon source also differs significantly between the host and symbionts within the shade-adapted corals (p = 0.009; PERMANOVA pairwise; Fig. 6). However, the carbon source did not differ between the host and symbionts of the light-adapted corals nor between the shade- and light-adapted hosts (p > 0.05; PERMANOVA pairwise; Fig. 6). A significant difference was also found in the nitrogen CSIA-AA, where the nitrogen isotope of the symbionts differed between the light- and shade- adapted corals, and between the host and symbionts of the shade-adapted corals (p = 0.008 and p = 0.027 respectively; PERMANOVA pairwise; Fig. 7). There was no significant difference in the nitrogen isotope of the light-adapted hosts compared to their symbionts, nor between the shade-adapted and light-adapted hosts (p > 0.05; PERMANOVA pairwise; Fig. 7). Using the nitrogen isotope of glutamic acid and phenylalanine to calculate the trophic position (TP) there was a significant difference in TP between the light- and shade- adapted corals, but not between hosts and symbionts (p = 0.034 and p > 0.05 respectively; PERMANOVA; Fig. 8). However, pairwise tests comparing TP between light- and shade- adapted symbionts and light- and shade- adapted hosts were not significant (p > 0.05; PERMANOVA pairwise; Fig. 8).

## Discussion

*Madracis pharensis* is a ubiquitous coral found across a broad geographic and vertical distribution^20^. In the Mediterranean, the species is documented from shallow reefs to the lower mesophotic (0–80 m). In the Bay of Haifa, we found individuals of *M. pharensis* on the surface of a small shipwreck at 30 m depth that showed visible differences in coloration (brown vs. white/pink) based on differential light conditions experienced due to orientation/location on the shipwreck (170PAR vs 106PAR). Based on molecular identification, we found that despite strong differences in phenotypes, these individuals were the same species of coral (*M. pharensis*). We also found that skeletal morphology, using basic polyp features, did not differ between corals living in the shaded overhang compared to those exposed to light. Thus, although the visual coloration of colonies in each orientation was in strong contrast, their molecular and morphological fingerprints were not. Similar skeletal morphologies under different light conditions contrast previous findings for other coral species, where skeletal morphology was shown to differ intraspecifically across light gradients^41–43^. These previous studies speculated that changes in skeletal morphology in response to light were an adaptive response that increased light harvesting capabilities to ensure sustained photosynthetic efficiency^8,44^. The lack of skeletal differences found in this study might suggest that these corals are less dependent on light-driven photosynthesis than the previously studied species, and thus modifications to skeletal morphology are not required.

Variations in light exposure are also known to affect the species of *Symbiodiniacaea* hosted by a coral, as certain symbiont species are documented to be more or less photosynthetically efficient under different light exposure scenarios either within^42^ or between different coral hosts^45^. In fact, several studies have documented shifts in associated symbionts across depth gradients for a variety of coral species, including *M. pharensis*^10,11,17,46–49^. Here, we found that individuals exposed to light primarily hosted *Breviolum psygmophilum *(clade B2), while those in the dark hosted a range of species dominated by *Symbiodinium microadriaticum *(clade A1), with *B. psygmophilum* and *Cladocopium* spp*.* in lower densities. Previous studies have found a higher diversity of symbiont species hosted by coral colonies that have experienced dysbiosis^6,50,51^. It is hypothesized that maintaining a diverse assemblage of symbiont species may assist in sustaining photosynthesis under variable conditions^52^. Shade-adapted *M. pharensis* colonies also had significantly reduced densities of photosymbionts and significantly lower concentrations of chlorophyll *a* compared to those oriented towards the light. While these differences in symbiont density and chlorophyll concentration would suggest reduced available photosynthate for the shade-adapted corals than the light-adapted counterparts, the total protein content for the shade-adapted corals was equal to that of the light-adapted corals, even slightly higher, suggesting that these corals sustain equivalent levels of protein.

These seemingly contradictory results might be explained by differences in light use efficiency. Here we found significant differences based on orientation for one photophysiology metric, the connectivity parameter (p), which was significantly higher in the light-adapted corals. The connectivity parameter defines the probability of the excitation energy transfer between individual photosynthetic units, photosystems I and II (PSI and PSII)^38^. Under natural diel light cycles, an increase in energy transfer (connectivity) is directly proportional to the functional absorption cross section of PSII, which is a measure of the probability that an absorbed photon will drive a photochemical reaction^53^, suggesting a common biophysical mechanism^54^. Gorbunov et al.^54^ propose that in the dark, such as the shade-adapted *M. pharensis* colonies in this study, connectivity between photosystem units is low and the thylakoid membrane units are energetically segregated^54^. The low connectivity parameter for the shade-adapted corals might suggest, therefore, that the photosynthetic pathway is uncoupled and does not result in the production of photosynthate. In high light environments, higher connectivity (energy transfer) between photosystems is expected as it promotes redistribution of excitons, thus providing protection from excess energy flux. However, we did not find a similar significant increase in the functional absorption cross section in response to orientation towards the light, which may suggest that, despite receiving 40% of available surface light, the amount of light reaching the surface of the wreck is below saturation.

All hermatypic corals are mixotrophic and, under laboratory conditions, some can survive on a purely autotrophic diet or when fed can be voracious predators, yet the extent to which corals rely on heterotrophy in the wild remains poorly understood^15,30,55,56^. Corals with a TP of 1 are assumed to rely only on autotrophy while corals with a TP > 1.5 are considered more heterotrophic. All individuals examined here were found to have a TP higher than 2, regardless of orientation, indicating that corals in both orientations are primarily heterotrophic, and again suggesting that overall light availability at the site is limited and may be insufficient to support a predominantly autotrophic diet. Importantly, these results highlight the capacity of this facultative symbiotic species to primarily utilize heterotrophy in low light environments. The facultative symbiotic coral *Oculina patagonica* has also been found to grow and survive for long periods of time under dark conditions in the laboratory and naturally in caves by relying on an exclusively heterotrophic diet^30,57,58^. Similarly, the relative contribution of autotrophy and heterotrophy were found to range among *Pocillopora meandrina* individuals living close to one another, further supporting trophic plasticity in corals^55^. Interestingly, the shade-adapted corals in this study had a significantly higher TP than the light-adapted individuals, indicating that they are even more reliant on heterotrophy and may be sustaining their symbionts. Many studies suggest that the host can regulate the transfer of photosynthate from the symbiont to the host, as well as nitrogen from the host to the symbiont^59–61^. When exploring the coral carbon source signature from the essential amino acids in this study, we found that in the light-adapted corals, the carbon source was the same for the host and the symbionts suggesting tight nutrient cycling. However, in the shade-adapted corals, the carbon signature differed between the host and the symbionts, indicating different sources of carbon. Likewise, the amino acid nitrogen isotope analysis showed a different nitrogen signature for symbionts based on orientation. Previous studies have shown that under short laboratory conditions, the symbionts are the first site of heterotrophic assimilation^62^. Since the coral host and symbiont are also known to share nutrients in both directions^62^ it would be expected that the hosts and symbionts will reach a steady state where they have a similar signature, which was not the case for the shade-adapted corals. Hence, the different signatures between the host and symbionts for the shade-adapted corals suggest unequal sharing of resources, where the symbiont is receiving nutrients obtained from ingested prey by the host^63^, but also has an alternative nutrient source that is not being shared. One potential alternative nutritional source for the shade-adapted symbionts could be amino acid resynthesis, which may alter the carbon signature of these symbionts compared to the host leading to the results found here^55^. Regardless of the alternative nutrient source, our results suggest that the host-symbiont relationship in the shade-adapted corals is more likely parasitic rather than mutualistic^64^. Another explanation might be that the symbionts are responsible for a metabolic pathway that we are unaware of, like that in the pea aphids-Buchnera symbiosis where the symbiont sustains itself but also produces byproducts that are beneficial for the host^65,66^.

Overall, we found that conspecific individuals living at the same location and depth but experiencing dramatically different light conditions had different consortia of symbiont species, differences in the probability of excitation energy transfer between photosynthetic units, and different amino acid isotopic signatures. These results all suggest that *M. pharensis* is highly plastic in its physiological response to light through a facultative symbiotic relationship. Thus, our findings highlight the capacity of corals to potentially switch to a predominantly heterotrophic diet when light availability and/or symbiont densities are too low to sustain sufficient photosynthesis. This potential metabolic plasticity may provide resilience for corals in the face of climate change, either through diet supplementation during periods of heat stress induced dysbiosis or through survival in low-light regions that may serve as thermal refuges.

## Methods

### Study site

This study took place in the Bay of Haifa, an industrial port located along the northern Mediterranean coast of Israel. The benthic habitat consists primarily of fields of macroalgae, sponges, ascidians, and interspersed scleractinian corals. Although coral diversity is relatively low, species identified in the region include *M. pharensis, O. patagonica, and Phylangia americana* (Morris Kahn Marine Research Station Long Term Ecological Research program [MKMRS LTER]; established in 2014). Due to a long history of shipping, several sunken vessels can be found in the region. The Leonid shipwreck is located towards the southern end of the bay (32.86975N 34.95405E) on a sandy bottom at 30 m depth (Fig. 1). It is assumed to have been a working ship that was stripped of all equipment before being sunk between 30 and 50 years ago. The boat is 42 m long and 11 m wide. The surfaces of the wreck are covered in algae, various invertebrates, and scleractinian corals. The vessel’s structure creates microhabitats so that organisms may be oriented towards the sunlight or under an overhang, resulting in substantial variations in light availability. Light levels at this site were measured using a HOBO light logger in September 2023 at 10am. During the dive, loggers were set at the same orientation as the corals. To convert Lux to µmol quanta m^−2^ s^−1^ (PAR), we compared HOBO measurements with apogee quantum meter (MQ-650 ePAR meter, Apogee Instruments Inc., USA). The light levels ranged from ~ 410 µmol quanta m^−2^ s^−1^ PAR at the water’s surface to ~ 172 µmol quanta m^−2^ s^−1^ PAR (42% of surface light) on the top side of the ship and ~ 106 µmol quanta m^−2^ s^−1^ PAR (25% of surface light) in the overhang environment. The water temperature at 30 m was 29 °C.

### Molecular identification

Small tissue biopsies (1cm^2^) were taken in November 2020 from *M. pharensis* colonies facing up (light-adapted) and facing down (shade-adapted) and preserved immediately in DNA/RNA shield (Zymo R1100) and frozen at − 20 °C prior to DNA extraction (n = 5). DNA was extracted using a Promega Wizard^®^ Genomic DNA Purification Kit following the manufacturer’s protocol. We used the highly conserved cytochrome oxidase subunit 1 (*COI*) using the following primers—FOL-LDEG (forward) 5′-TCWACHAAY CAT AAR GAY ATWGG-3′ and FOL-HDEG (reverse) 5′-TCWACHAAY CAT AAR GAY ATWGG-3′ (modified from^67^). In addition, the internal transcribed spacer (*ITS2*) region of *Symbiodiniaceae* rDNA was amplified using *Symbiodiniaceae*-specific primers CS1F (forward) 5′-ACA CTG ACG ACA TGG TTC TAC ATG TGA ATT GCA GAA CTC CGT G-3′ and CS2R (reverse) 5′-TAC GGT AGC AGA GAC TTG GTC TTA CTT ATA TGC TTA AAT TCR GCGG-3′ taken from Arif et al.^68^. The host *COI* region was sequenced using the Sanger sequencing method using the ABI 3730xl DNA Analyser while *Symbiodiniaceae ITS2* was sequenced on the Illumina Miseq using a v2-500 cycle kit to generate 250 × 2, paired-end reads. *Symbiodiniaceae ITS2* data were demultiplexed by the Illumina software, and the demultiplexed fastq files were further analyzed. The resulting *COI* sequences (n = 2 per orientation) were aligned using ClustalW to create a consensus sequence, which was blasted in NCBI’s GenBank for species identification. In addition, paired forward and reverse fastq.gz files (n = 5 light-adapted; n = 3 shade-adapted) were submitted to SymPortal^69^ to assess the diversity and relative abundance of symbiont species within each sample. The evolutionary history of the coral host species was inferred using the Maximum Likelihood Method and Tamura-Nei model. The tree with the highest log likelihood (− 1584.38) is shown using the software MEGA X.

### Skeletal morphology

Skeletal fragments from tissue biopsies (November 2020) were used to assess differences in polyp morphology based on orientation (shade vs. light; n = 5). Imaging was done using scanning electron microscopy (ZEISS SigmaTM SEM, Germany) by using an in-lens detector (5 kV, WD = 1.5–2.5 mm) on 3–19 polyps per colony, and resulting images measured for calyx width, columella width, and septa width. Data for each metric met the assumptions of normality (Levene’s test) and equal variance (Shapiro–Wilk test) and were analyzed for differences based on orientation using Student’s t-tests.

### In -situ physiological analyses

In situ physiological assays were conducted 18 July 2022 on *M. pharensis* colonies representing each orientation (light, n = 7; shade, n = 10) using a Diving-Fluorescence Induction and Relaxation (FIRe) fluorometer^32^, following methods described by Carpenter et al.^70^. In brief, to account for intra-colony differences in light exposure, five readings were collected per colony and averaged to reflect the photophysiology of the entire colony in the analysis. The Diving-FIRe instrument is programmed to make measurements in two regimes—the first one in dark, and the second under saturating irradiance. The first sampling protocol is used to derive Fv/Fm, σPSII, the connectivity parameter (p) as well as the kinetics of quinone re-oxidation in a dark-adapted state. The second protocol is used to derive the absolute maximum ETR (ETRmax) achievable at saturating PAR. In the Diving-FIRe, ETRmax is calculated from the kinetics of quinone re-oxidation recorded under saturating PAR (= ca. 3 × Ek) as described in Ref.^38^. As Diving-FIRe is based on a kinetic approach, it offers instantaneous measurements of ETRmax without dark adaptation of the sample therefore allowing *in-situ* measurements during the daytime.

Variance and normality of photophysiology metrics were assessed using Shapiro Wilk tests, histograms, and residual QQ plots. Photosynthetic efficiency (F_v_’/F_m_’) required no transformation to meet the assumptions and was analyzed by orientation (light vs. shade) using a one-way ANOVA. The connectivity parameter (p) was transformed by taking the fourth root, σ_PSII_ was transformed by taking the square root of the log, and energy transfer (TauAv1) was transformed by taking the sixth root. All transformed data were compared by orientation using a one-way ANOVA. P_max_ did not meet the assumptions even after transformation and therefore the untransformed data were examined by orientation using a non-parametric Kruskal–Wallis test.

### Ex situ physiological analyses

Samples from five *M. pharensis* colonies from each orientation (light vs. shade) were collected in September 2022 and transported to the laboratory at the University of Haifa for analysis of symbiont density, chlorophyll concentration, and protein content. Each coral fragment was airbrushed into a sterile ziplock bag containing 4 mL distilled water (DW). The tissue slurry was transferred to 15 mL centrifuge tubes and electrically homogenized for 20 s before centrifugation at 5000xg for 10 min at 4 °C. A 100μL aliquot of supernatant was taken to determine animal host protein concentration using the QPRO-BCA kit standard (Cyanagen, Italy) following the manufacturer protocol. A PerkinElmer (2300 EnSpire R, United States) plate reader was used to determine the total protein concentration at a 562 nm emission wavelength. Following a further centrifugation at 5000 × *g* for 5 min, removal of the supernatant, and resuspension in filtered seawater, aliquots of 50µL of the homogenate were used to determine algal symbiont density by fluorescent microscopic counts using a hemocytometer (n = 4 per sample). Each replicate was photographed both in brightfield and in fluorescent light using 440 nm emission to identify chlorophyll and ensure counting of symbiont cells only. The resulting cell counts were normalized to average host protein concentration for each fragment. Chlorophyll *a* (chl *a*) concentrations were measured in 2 ml of homogenate that was incubated overnight with 90% cold acetone at 4 °C. After incubation, the samples were centrifuged at 5000xg for 5 min at 4 °C, and sample absorbance was determined in a 96-well plate using the equations of Jeffrey and Humpfrey^71^, with path length adjusted to 0.555 cm (200μL sample volume well^−1^).

Algal cell density (cells/ug protein) did not meet the assumptions of normality and variance and were transformed by taking the log + 1, and chlorophyll density (ug chl a/ µg protein) was square root transformed. Chlorophyll per symbiont cell (ug/cell) and protein content (µg/ml) met the assumptions of normality and variance. All data were analyzed by orientation using a one-way ANOVA.

### CSIA-AA

The nitrogen and carbon isotopic compositions of amino acids were determined by gas chromatography/combustion/isotope ratio mass spectrometry (GC/C/IRMS). Tissue slurry and symbionts from samples collected in November 2020 were lyophilized. Approximately 4 mg of dry tissue and symbiont were hydrolyzed with 6nml HCl at 150 °C for 70 min^72^. The acid-hydrolyzed host and symbiont samples were first derivatized using the Ezfaast kit before isotopic analysis was performed, according to Martinez et al.^15^. Briefly, 4 mg of the hydrolyzed samples was derivatized with the Ezfaast kit with a slight modification of replacing reagent 6 with dichloromethane as solvent. Amino acids were separated on a Zebron ZB-50 column (30 m, 0.25 mm, and 0.25 µm) on a Thermo Scientific Trace 1300 Gas Chromatograph using helium as the carrier gas at a constant flow of 1.5 ml/min. For carbon analysis, 1.5 µl was injected in split mode (1:15) at 250 °C, while 2 µl was injected in splitless mode at 250 °C for nitrogen analysis. The separated amino acids were split on the MicroChannel device into two direction flows: Thermo Scientific ISQ quadruples for amino acid identification and Thermo Scientific Delta-V advantage for C and N isotope analysis. To determine the isotopic ratio of carbon and nitrogen, the separated amino acids were combusted in a Thermo scientific GC isolink II at 1000 °C for CO_2_ and N_2_. Before the sample was run into the Delta-V for N_2_ analysis, it passed through a cold trap with liquid nitrogen to freeze all other gasses. Duplicates for carbon and triplicates for nitrogen were injected from each sample. Stable isotope ratios were expressed in standard *δ* notation, with the standard for carbon being Vienna PeeDee Belemnite (VPDB) and for nitrogen atmospheric N_2_ (air). To account for carbons incorporated during the derivatization process, we followed the correction factor of Docherty et al*.*^73^ for each amino acid. The trophic position (TP) was calculated using glutamic acid and phenylalanine with the predefined equation of Chikaraishi et al*.*^40^ with the constants from Martinez et al*.*^15^.

## Data Availability

All raw data and code are available on Zenodo at 10.5281/zenodo.10055775.
